# Pregnancy-Related Heart Disease in the Emergency Department

**DOI:** 10.3390/jpm15040148

**Published:** 2025-04-08

**Authors:** Nicole R. Hodgson, Rachel A. Lindor, Jessica Monas, Kimberly Heller, Patrick Kishi, Aaron Thomas, Cody Petrie, Lauren B. Querin, Andrej Urumov, David S. Majdalany

**Affiliations:** 1 Mayo Clinic Department of Emergency Medicine, Phoenix, AZ 85054, USA; monas.jessica@mayo.edu (J.M.); heller.kimberly@mayo.edu (K.H.); kishi.patrick@mayo.edu (P.K.); thomas.aaron@mayo.edu (A.T.); petrie.cody@mayo.edu (C.P.); querin.lauren@mayo.edu (L.B.Q.); urumov.andrej@mayo.edu (A.U.); 2 Mayo Clinic Department of Emergency Medicine, Rochester, MN 55905, USA; lindor.rachel@mayo.edu; 3 Mayo Clinic Department of Cardiovascular Diseases, Phoeniz, AZ 85054, USA

**Keywords:** pregnancy-related cardiac emergencies, cardiovascular changes in pregnancy, adult congenital heart disease in pregnancy

## Abstract

Pregnancy induces significant physiologic changes that impact the cardiovascular system, potentially exacerbating pre-existing cardiac conditions or precipitating new illnesses. Pregnant patients with cardiac emergencies pose unique challenges, as standard interventions may pose risks to the developing fetus. This article aims to enhance emergency physicians’ confidence in managing pregnancy-related cardiac emergencies by providing a structured approach to initial evaluation and stabilization. We review eight common categories of pregnancy-associated cardiac illness: gestational hypertension and pre-eclampsia, cardiomyopathy, arrhythmias, valvular disease, aortopathies, congenital heart disease and pulmonary hypertension, coronary disease, and anticoagulation-related complications. For each condition, we summarize relevant pregnancy-specific pathophysiology and outline evidence-based, personalized emergency management strategies.

## 1. Introduction

Multiple physiologic changes occur during pregnancy that alter the cardiovascular system. Increases in cardiac output, circulating blood volume, and heart rate, along with pro-thrombotic changes, may exacerbate or reveal pre-existing cardiac issues or may cause new illnesses to develop.

Pregnant patients with cardiac illness presenting to the emergency department (ED) may unnerve even the most seasoned emergency physician. Diagnostics and treatments used in non-pregnant patients may be harmful to a developing fetus, leading to clinician uncertainty when timely intervention is crucial. Although pregnancy-related cardiac emergencies are best handled with a multidisciplinary team, including high-risk obstetricians and cardiologists, these specialties are unlikely to be immediately available in the ED setting. Awareness of common ED presentations and initial disease management is therefore necessary for the stabilization of pregnant patients with cardiac emergencies.

In this article, we discuss eight common categories of pregnancy-related cardiac illness, including gestational hypertension and pre-eclampsia, cardiomyopathy, arrhythmias, valvular disease, aortopathies, congenital heart disease and pulmonary hypertension, coronary disease, and anticoagulation issues and bleeding. We briefly outline the anatomy and physiology of each disease in pregnancy before discussing disease-specific personalized ED management, hoping to increase the comfort of emergency physicians treating critical cardiac illness in pregnancy. We include Table 1 below as a quick reference for readers to use while treating patients in real time.

## 2. Discussion

### 2.1. Gestational Hypertension and Pre-Eclampsia

#### 2.1.1. Anatomy and Physiology

Elevated blood pressure in pregnancy is increasingly common, multifactorial, not fully understood, and may be indicative of one of several related but distinct diagnoses. Gestational hypertension is diagnosed in patients who meet the following criteria with resolution within 12 weeks of delivery: new onset of systolic blood pressure ≥ 140 and/or diastolic BP ≥ 90 after 20 weeks of gestation, normal urine protein excretion in pregnancy, and no signs of end-organ dysfunction. About half of these patients develop pre-eclampsia [1]. Risk factors for the development of pre-eclampsia include advanced maternal age, the use of assisted reproductive technology, obesity, diabetes, multifetal gestations, chronic hypertension, a history of pre-eclampsia in a previous pregnancy, renal disease, sleep apnea, and several autoimmune conditions; however, the majority of cases of pre-eclampsia arise in healthy nulliparous women [1]. Some patients deemed to have a higher risk of progression to pre-eclampsia, such as those with advanced maternal age, may be started on low-dose aspirin empirically. Pre-eclampsia is estimated to affect 2–8% of global pregnancies [1].

Pre-eclampsia, understood as a progressive disorder related to abnormal placental and maternal vascular dysfunction, differs from gestational hypertension as it is accompanied by proteinuria or end-organ dysfunction. Signs of end-organ dysfunction include thrombocytopenia (platelets < 100,000), creatinine increase (>1.1 mg/dL or doubling the baseline level), an elevation in liver transaminases (twice the upper limit of normal), new persistent headache not accounted for by other causes, pulmonary edema, or visual changes. Patients may progress to develop pre-eclampsia with severe features, identified by pressures > 160/110 mm Hg or certain combinations of end-organ dysfunction, including HELLP syndrome, named for the co-occurrence of Hemolysis, Elevated Liver enzymes, and Low Platelets with or without hypertension. Approximately 2–3% of patients with pre-eclampsia progress to eclampsia and experience a tonic–clonic seizure or coma without another cause, often in the post-partum period [1].

#### 2.1.2. ED Presentation

Elevated blood pressure in pregnancy should be noted in any patients presenting to the ED, especially those at or after 20 weeks of gestation. Hypertension in pregnancy should be treated with antihypertensives, with methyldopa, labetalol, nifedipine, and hydralazine as preferred first-line agents. Patients with elevated pressures should be screened for pre-eclampsia based on both symptoms as well as laboratory analyses. Any findings of end-organ dysfunction could prompt expedited delivery or medical optimization from the patient’s obstetrics team, depending on gestational age. For example, some patients would benefit from taking aspirin, magnesium, or antihypertensives, while others may require inpatient admission or urgent delivery [2].

In patients at risk for eclampsia, most patients are hypertensive on presentation. The most common preceding symptoms experienced by patients include headache, vision changes, and right upper quadrant pain [3].

#### 2.1.3. Management

Eclampsia represents the most emergent end of the spectrum of hypertension in pregnancy. In patients who are identified as high risk or who progress to seizure, several steps can help reduce morbidity and mortality. Magnesium sulfate is the most effective seizure prophylaxis in this group of patients, administered at a dose of 6 g over 15–20 min [4]. During a seizure, gravid patients should be rolled onto their left side to decompress the inferior vena cava, and supplemental oxygen should be administered to lessen hypoxia from hypoventilation. Antihypertensives such as labetalol or hydralazine should be administered to reduce the risk of stroke, which accounts for up to 20% of mortality in patients with eclampsia. After the resolution of the seizure, a loading dose of magnesium sulfate should be given to prevent additional seizures, and for seizures that have not resolved after five minutes, a benzodiazepine should be administered. For eclampsia, as well as related disorders, such as HELLP syndrome, the definitive treatment is delivery [1].

### 2.2. Peripartum and Postpartum Cardiomyopathy

#### 2.2.1. Anatomy and Physiology

Peripartum/postpartum cardiomyopathy (PPCM) is a form of idiopathic dilated cardiomyopathy that manifests in the last month of pregnancy or within five months postpartum. Patients must have the absence of another identifiable cause of heart failure or structural heart disease before the last month of pregnancy [5]. Classic echocardiographic criteria include a reduced left ventricular ejection fraction (LVEF < 45%), reduced fractional shortening (<30%), or dilated left ventricular end-diastolic dimension (LVEDD > 60 mm) [5,6].

The current incidence of PPCM in the United States is 1 in 4000 live births [5,7]. Identified risk factors include African American descent, older maternal age, multifetal pregnancies, and hypertensive disorders during pregnancy [5,7,8,9].

The pathophysiology of PPCM remains incompletely understood but is likely multifactorial. Elevated oxidative stress of pregnancy and prolactin cleavage into a cardiotoxic 16-kDa prolactin fragment (vasoinhibin) have been implicated in myocardial injury [5,6,8]. Additionally, vascular endothelial growth factor suppression, heightened inflammatory cytokines, and genetic predisposition likely contribute to myocardial dysfunction [8].

#### 2.2.2. ED Presentation

PPCM patients present with typical heart failure symptoms. Dyspnea with exertion, orthopnea, paroxysmal nocturnal dyspnea, and lower extremity edema are hallmark clinical manifestations. An electrocardiogram (ECG) may reveal sinus tachycardia or nonspecific ST-T wave changes, and chest radiography can demonstrate pulmonary congestion, cardiomegaly, and pleural effusions. Initial workup should include a point-of-care ultrasound (POCUS) transthoracic echocardiogram (TTE), which will show a reduced left ventricular systolic function (LVEF < 45%) or left ventricular dilation. Laboratory values should include NT-proBNP and troponins. 

#### 2.2.3. Management

The primary goal in the ED is hemodynamic stabilization and symptom relief. Standard heart failure management applies, with loop diuretics (furosemide) for volume overload and vasodilators (hydralazine and nitrates) to reduce afterload while maintaining uteroplacental perfusion. ACE inhibitors and angiotensin II receptor blockers should be avoided in pregnancy due to fetal risks. Inotropes may be necessary for patients with cardiogenic shock, and mechanical circulatory support should be considered in refractory cases. Anticoagulation with heparin is recommended, given the increased risk of thromboembolism due to left ventricular dysfunction and hypercoagulability in the peripartum state.

The goals for the definitive management of PPCM are symptom control, cardiac function improvement, and prevention of thromboembolism and ventricular arrhythmias. Prognosis varies widely, with mortality rates depending on geographic region, but averaged at 15% worldwide [5,6,9,10]. Patients at higher risk for complications include those with QT interval prolongation, LVEF < 30%, LVEDD > 60 mm, and biventricular dysfunction [6]. PPCM patients with LVEF < 30% at the time of diagnosis are at risk for sudden cardiac death secondary to arrhythmias, and a wearable cardioverter–defibrillator is recommended [5].

### 2.3. Arrhythmias

#### 2.3.1. Anatomy and Physiology

Pregnancy is associated with an increased risk of arrhythmia [11,12]. This is attributed to structural, autonomic, and hormonal changes, including increased sympathetic activity leading to higher circulating catecholamines, intravascular volume expansion, myocardial stretch, and increased heart rate and cardiac output [13]. Risk factors include advanced age, pre-existing hypertension, diabetes mellitus, obesity, and cardiovascular disease, including previous arrhythmia and congenital heart disease [12,14,15]. Although the exact incidence of arrhythmia in pregnancy is unknown, one study examining pregnancy-related hospitalizations found that pregnant women experience any arrhythmia at a rate of 68 per 100,000 admissions [11].

ECG alterations occur in normal pregnancy, including shortening of the PR interval and prolongation of QTc [13]. These values typically remain within the normal range; however, women predisposed to repolarization abnormalities carry increased risk. 

#### 2.3.2. ED Presentation

The most common arrhythmias seen in pregnancy are atrial tachycardias, including supraventricular tachycardia (SVT), atrial fibrillation, and atrial flutter [12,15]. Ventricular arrhythmias are rare but are seen more commonly in women with congenital heart disease or underlying cardiomyopathy [14]. Other than sinus bradycardia, conduction disorders and bradyarrhythmias are uncommon [16]. Pregnant patients with arrhythmia present similarly to non-pregnant individuals, which varies depending on the type of arrhythmia and underlying comorbidities. Patients may be asymptomatic or present with palpitations, syncope, chest pain, dyspnea, or even cardiac arrest. 

#### 2.3.3. Management

Pregnant patients with arrhythmia carry an increased risk of maternal and fetal complications [12,15]. Early detection and management is vital.

##### Supraventricular Tachycardia (SVT)

In stable SVT, first-line therapies include vagal maneuvers, followed by adenosine. In the absence of pre-excitation, second-line agents include beta-blockers or calcium channel blockers. Flecainide or procainamide are acceptable. If unstable, synchronized cardioversion with fetal monitoring is indicated [12,17].

##### Atrial Fibrillation/Atrial Flutter

In stable patients, first-line rate control therapy is beta-blockers. Second-line agents include digoxin or calcium channel blockers. Flecainide or sotalol may be used for rhythm control, but amiodarone should be avoided. If unstable or refractory, anticoagulation with cardioversion is indicated. Patients should typically be anticoagulated for at least 4 weeks after cardioversion, especially if the arrhythmia onset time is unknown; decisions to continue anticoagulation should be dictated by the CHA_2_DS_2_VASc score, as in non-pregnant patients. Direct oral anticoagulants are contraindicated in pregnancy, and alternative strategies such as low-molecular-weight heparin and warfarin (after the first trimester) should be used [12]. 

##### Ventricular Tachycardia (VT)

If an underlying structural heart disease is present, treatment is targeted at the underlying condition. The first-line treatment in stable acute VT in pregnancy is lidocaine. Second-line agents are procainamide or quinidine. Magnesium sulfate is used in cases of torsade de pointes or polymorphic VT. If unstable, cardioversion is indicated [12,14].

##### Cardiac Arrest

Resuscitation should follow standard Advanced Cardiovascular Life Support protocols, including medications, doses, and defibrillation protocols. Lateral displacement of the uterus is indicated, and resuscitative hysterotomy may be considered [12]. 

### 2.4. Valvular Disease

#### 2.4.1. Anatomy and Physiology

Physiological increased cardiac output during pregnancy often leads to increased flow and increased gradients through valvular lesions. Valvular diseases in pregnant women are typically congenital, with between 1 and 2% of reproductive-age women having valvular heart disease [18], but can also be due to rheumatic and acquired causes. Due to the hypercoagulable state of pregnancy, patients are at increased risk of thrombosis, especially in those with prosthetic valves [19,20,21,22].

Left-sided obstructive valvular lesions such as mitral and aortic stenosis are associated with the highest risks and are poorly tolerated in pregnancy [18,20,22]. Mitral stenosis, typically from rheumatic heart disease, is the most common cause of maternal death from cardiac causes globally [22]. In contrast, regurgitant valvular lesions are usually tolerated in pregnancy due to the decrease in afterload and systemic vascular resistance seen in physiologic pregnancy changes.

#### 2.4.2. ED Presentation

Worsening left-sided obstructive valvular lesions can lead to complications, including pulmonary edema, atrial and ventricular arrhythmias, cerebrovascular events, and death [22,23]. Pregnant patients with mechanical valves are at increased risk for thrombosis, stroke, heart failure, and hemorrhage [22]. Patients may present to the ED with symptoms of these complications, such as shortness of breath, chest pain, palpitations, syncope, fluid retention, neurological deficits, and acute decompensation in hemodynamic stability.

#### 2.4.3. Management

The management of valvular disease in pregnancy depends on the lesion and degree of severity. ED POCUS TTE should be considered, especially in hemodynamically unstable patients. Based on the underlying cause, medical management of valvular disease in the ED can include the use of nodal-blocking agents, diuretics, anticoagulation, and vasodilators. Ultimately, treatment should be managed with a multidisciplinary approach with high-risk obstetricians and cardiologists [18].

### 2.5. Aortopathies

#### 2.5.1. Anatomy and Physiology

Pregnancy induces significant changes in the connective tissues of the pelvic region and the rest of the body to accommodate fetal growth, physiologic changes, and preparation for labor. These changes involve alterations in the biochemical composition and mechanical properties of tissues such as the cervix, vagina, myometrium, and pubic symphysis [24]. This extends beyond pelvic organs to include blood vessels and the cardiovascular system. These are generally related to fluctuations in estrogen, progesterone, human chorionic gonadotropin (hCG), prolactin, corticotropin-releasing hormone (CRH), and insulin-like growth factor I (IGF-I) [25].

Estrogen is the primary hormone that plays a crucial role in cardiovascular adaptation associated with pregnancy. It leads to endothelial relaxation via vasodilators, including nitric oxide [26], resulting in vascular remodeling characterized by increased luminal diameter and wall thickness in order to accommodate the increased cardiac output and blood volume associated with pregnancy [27]. Additionally, up-regulation of angiotensin receptors in the renin–angiotensin system leads to decreases in blood pressure and vascular resistance [28]. The ultimate result of these physiologic changes is progressive aortic dilation and increased compliance of the aorta and other large blood vessels. These changes are most prominent in the third trimester but extend up to 12 weeks postpartum.

#### 2.5.2. ED Presentation

Acute aortic emergencies during pregnancy include aortic dissection, atherosclerotic ulcer, and intramural hematoma. The most significant risk factor for these conditions is underlying connective tissue disorder [29]. The American College of Cardiology (ACC) and the American Heart Association (AHA) recommend that women with known aortopathy undergo pre-pregnancy counseling, genetic testing, and aortic imaging. Aortic dissection in pregnancy remains rare, affecting 0.69 per 100,000 pregnancy-related hospitalizations [30].

Acute aortic emergencies most commonly present with a sudden onset of severe chest or back pain occasionally radiating to the abdomen, sometimes described as “tearing” or “ripping”. The abrupt maximal onset of pain, compared to the crescendo-type pattern, can distinguish aortic involvement from acute coronary syndrome. Physical exam findings of asymmetric blood pressure between limbs, aortic regurgitation murmurs, pulse deficits, and signs of end-organ hypoperfusion, including shock and neurologic deficits, are signs of extremis. Untreated acute aortic syndrome has extremely high mortality with rapidly increasing rates of 1–2% per every hour of missed management [31,32]. 

#### 2.5.3. Management

Intravenous access and cross-sectional imaging are required emergently. Prompt surgical and obstetric consultation while managing blood pressure and preparing for transfusion are essential. For less acute situations, the Aortic Dissection Detection Risk Score (ADD-RS) is a validated stratification tool that incorporates the history of high-risk conditions, symptoms, and exam findings to suggest a role for D-dimer testing versus proceeding directly to cross-sectional imaging in the workup of suspected aortic emergency [33].

For acute type A aortic dissections in the first or second trimester, urgent aortic surgery with fetal monitoring is recommended; in the third trimester, emergent cesarean delivery precedes aortic surgery. For acute type B aortic dissections, medical therapy is recommended unless there are indications of extremis requiring surgical intervention. Beta-blockers and anti-hypertensives are used to control heart rate and blood pressure with a goal systolic blood pressure of less than 120 mmHg, with first-line medications, including esmolol and labetalol. Progressive aortic dilation during pregnancy in the absence of concerning symptoms or presentation may still be an indication for prophylactic aortic surgery, often endovascular in nature [31,34]. Cesarean section should be considered once aortic dimensions reach 45 mm.

### 2.6. Congenital Heart Disease and Pulmonary Hypertension

#### 2.6.1. Anatomy and Physiology

Pregnancy induces hemodynamic changes, including a 30–50% increase in intravascular volume and cardiac output, a peak in the third trimester due to increased stroke volume, and a 10–20 bpm rise in heart rate. These changes can exacerbate right ventricular strain in congenital heart disease (CHD)-associated pulmonary hypertension (PH), myocardial dysfunction, or valvular disease, increasing the risk of heart failure [35].

In CHD-associated PH, chronic right ventricular pressure overload leads to hypertrophy, dysfunction, and failure. Septal defects can cause left-to-right shunting, worsening pulmonary blood flow, and vascular remodeling. Persistent PH may progress to Eisenmenger syndrome, characterized by right-to-left shunting, hypoxemia, and cyanosis, historically linked to 50% maternal mortality, though outcomes have improved with modern management [36]. The hypercoagulable state of pregnancy increases the thromboembolic risk to 2% in CHD pregnancies compared to 0.05–0.10% in normal pregnancies [37].

#### 2.6.2. ED Presentation

Patients with CHD and PH commonly present with dyspnea, chest pain, palpitations, or syncope, suggesting PH progression, right ventricular failure, or arrhythmias. Cyanosis, jugular venous distension, edema, and hepatomegaly indicate right heart dysfunction, while hypoxemia (<85%) and digital clubbing in Eisenmenger syndrome strongly predict fetal loss [35]. Tachycardia, hypotension, and hypoxemia require urgent evaluation.

BNP or NT-proBNP levels help assess right ventricular strain, while elevated troponins correlate with disease severity and mortality risk. Arterial blood gases may show respiratory alkalosis, while elevated lactate suggests poor perfusion. Echocardiography remains the gold standard for evaluating right ventricular function and pulmonary pressures, with bubble contrast useful for detecting right-to-left shunting [38]. ECG findings may include right axis deviation, right ventricular hypertrophy, or ST-T wave changes. Chest radiography may show cardiomegaly and pulmonary congestion, while CT pulmonary angiography is indicated when pulmonary embolism is suspected.

Pregnant women with cyanotic CHD face a high risk of adverse fetal outcomes, including preterm birth (45%) and fetal loss (12%), due to maternal hypoxemia, right-to-left shunting, and reduced uteroplacental perfusion [35]. The severity of maternal cyanosis (oxygen saturation < 85%) strongly correlates with fetal growth restriction and demise [39].

#### 2.6.3. Management

Emergency management should focus on hemodynamic stabilization. Oxygen therapy is crucial for maternal and fetal oxygenation, particularly in cyanotic CHD. Phenylephrine is the preferred agent for hypotension, especially in patients with systemic outflow tract obstruction; vasodilators should be avoided to prevent right ventricular decompensation [40,41]. Diuretics such as furosemide may be used with caution, but excessive diuresis can reduce preload and precipitate circulatory collapse [42]. Continuous cardiac monitoring is needed due to the high risk of arrhythmias and right heart failure. In PH crises, pulmonary vasodilators such as inhaled nitric oxide and prostacyclins may be required [43]. Anticoagulation should be individualized, particularly in patients with prosthetic valves, Fontan circulation, or atrial arrhythmias, to balance thromboembolism and bleeding risks [44].

A multidisciplinary team, including cardiology, maternal–fetal medicine, critical care, anesthesiology, pulmonary hypertension team, and neonatology, is essential for optimizing outcomes. Cesarean delivery is preferred in severe PH and Eisenmenger syndrome, while vaginal delivery may be considered with strict hemodynamic monitoring [45,46]. Regional anesthesia is favored to mitigate systemic vasodilation, though hypotension must be carefully managed [41,45]. The postpartum period carries the highest maternal mortality risk, with most deaths occurring within the first week, necessitating ICU-level monitoring and early intervention [47]. Fetal complications occur at over two and a half times the rate of the general population, requiring close neonatal surveillance [48]. Given the high maternal and fetal risks associated with CHD and PH, emergency medicine plays a critical role in early recognition of deterioration, hemodynamic stabilization, and timely multidisciplinary coordination to improve outcomes.

### 2.7. Coronary Artery Concerns

#### 2.7.1. Anatomy and Physiology

Acute coronary syndromes (ACS), including acute myocardial infarction (AMI), spontaneous coronary artery dissection (SCAD), primary coronary thrombosis, and coronary spasm, despite being rare occurrences during pregnancy [49], are associated with significant morbidity and mortality. Pregnant women are three to four times more likely to experience ACS compared to age-matched non-pregnant individuals [49,50,51], experiencing ACS in 1.7 to 6.2 out of every 100,000 deliveries [49].

Nonatherosclerotic causes are a major contributor to AMI in pregnancy [50]. However, atherosclerosis, driven by increasing maternal age and a higher prevalence of comorbidities, is an increasing risk factor [51]. Pregnancy-specific physiological changes contribute to the heightened risk of ACS. These include a 50% increase in blood volume and cardiac output, an elevated basal heart rate, physiological blood dilution, and iron deficiency [51]. The increased oxygen demand during the peripartum period, hemodynamic fluctuations, and the prothrombotic state associated with pregnancy further exacerbate the risk of ACS. Hemodynamic instability and connective tissue disorders are believed to increase susceptibility to SCAD, particularly in the late third trimester and peripartum period.

#### 2.7.2. ED Presentation

The presentation of ACS during pregnancy closely resembles that observed in the general population, manifesting with an acute onset of chest pain, dyspnea, syncope, and, in extreme cases, cardiac arrest [49,50,51]. ED assessment prioritizes obtaining a pertinent history and physical examination, supplemented by rapid diagnostic testing, including ECG and POCUS TTE, to evaluate regional wall motion abnormalities and cardiac biomarkers. Physiological ECG changes associated with pregnancy must be taken into account, such as left-axis deviation, non-specific repolarization abnormalities, inferior Q waves, and T-wave inversions [49,50,51].

#### 2.7.3. Management

Management of AMI in pregnancy primarily involves pharmacotherapy and coronary angiography. Thrombolysis is relatively contraindicated [51,52]. Pharmacologic treatment includes aspirin and heparin, both of which are considered generally safe for use during pregnancy [52]. However, caution is warranted when administering aspirin beyond 32 weeks of gestation due to the risk of premature closure of the ductus arteriosus [49,50]. Heparin, which does not cross the placenta, is not associated with adverse fetal effects [49]. When dual antiplatelet therapy is required, clopidogrel is the preferred agent and is administered for the shortest duration of time possible [52,53].

The indications for coronary angiography to guide percutaneous coronary intervention (PCI) in pregnant patients with ACS are comparable to those in non-pregnant individuals [52]. Concerns regarding fetal exposure to ionizing radiation should not preclude the procedure when clinically indicated [50,52]. PCI is recommended for atherosclerotic and thrombotic events, such as STEMI [50]. Management of SCAD is often conservative. PCI is of limited efficacy in cases of SCAD due to the inherent fragility of the affected blood vessels, which often results in poor revascularization outcomes as well as procedural complications [49,50,51,54].

A multidisciplinary approach involving cardiology and maternal–fetal medicine specialists is essential for optimizing the management of pregnant patients presenting with ACS, ensuring that the most appropriate and individualized therapeutic strategy is employed [51,53].

### 2.8. Anticoagulation Issues and Bleeding

#### 2.8.1. Anatomy and Physiology

Pregnancy-related bleeding complications range from hypercoagulable conditions like HELLP syndrome and disseminated intravascular coagulation (DIC) to hemorrhagic events such as antepartum and postpartum hemorrhage. Pregnancy induces a hypercoagulable state to maintain placental function and reduce postpartum hemorrhage risk [55,56]. This hypercoagulable state occurs due to a variety of changes. This includes an increase in procoagulant factors VII, VIII, IX, X, and XII, as well as fibrinogen and von Willebrand factor (VWF) [55,57,58]. Thrombin generation is enhanced due to an increase in prothrombin fragments PF1 and PF2. Additionally, anticoagulant activity decreases due to lower Protein S levels and resistance to activated Protein C [55,57]. Impaired fibrinolysis occurs due to the increased levels of plasminogen activator inhibitors PAI-1 and PAI-2, though fibrinolytic markers like D-dimer rise during pregnancy [58]. Mechanical changes, including venous stasis and endothelial alterations, further contribute to hypercoagulability [55,56,57].

#### 2.8.2. ED Presentation

These physiologic changes can predispose pregnant individuals to thrombotic, microthrombotic, and bleeding disorders. The most common thrombotic event is venous thromboembolism (VTE), with a four- to fivefold increased risk of deep vein thrombosis (DVT) and pulmonary embolism (PE) compared to non-pregnant women, occurring in 1–2 per 1000 pregnancies [59]. Notably, the risk of VTE continues into the postpartum period and is greatest in the first three weeks after delivery [59,60]. Risk factors for VTE in pregnancy include prior pregnancy-related VTE, hyperemesis (due to resultant dehydration), immobility, obesity, and thrombophilias [59]. There are multiple microthombotic disorders that can occur in pregnancy, the most common of which are pre-eclampsia and HELLP syndrome [61,62]. Among platelet disorders, gestational thrombocytopenia is the most common and accounts for approximately 75% of thrombocytopenia cases [63]. Immune thrombocytopenic purpura (ITP) and thrombotic thrombocytopenic purpura (TTP) are less common but also occur with increased incidence in pregnancy [64,65]. Antepartum and postpartum hemorrhage are potential bleeding complications in pregnancy, with an overall incidence of approximately 1–3% [66,67]. Hemorrhagic complications are associated with varied risk factors, including coagulopathies, advanced maternal age, and pre-eclampsia [68,69].

#### 2.8.3. Management

Managing coagulopathies and bleeding in pregnancy requires prompt recognition and targeted diagnostics. VTE diagnosis involves imaging—compression ultrasonography for DVT and CT angiography or ventilation–perfusion scanning for PE—with consideration of fetal risks. Low-molecular-weight heparin is preferred over unfractionated heparin for anticoagulation in the pregnant population due to a lower risk of adverse events [70]. For acquired bleeding disorders, appropriate diagnosis is key and involves laboratory testing, including platelet count, prothrombin time, partial thromboplastin time, and fibrinogen levels [71,72]. Management is unique to each condition. For antepartum and post-partum hemorrhage, a multi-disciplinary approach is recommended involving emergency physicians, obstetricians, and hematologists [73]. With hemorrhage in pregnancy, start with an assessment of hemodynamic stability, estimated blood loss, and determining the cause of bleeding. Common causes include retained products of conception, uterine atony, abnormal placentation, and underlying coagulopathies. Management may include the transfusion of mixed blood products, use of uterotonics such as oxytocin or methylergonovine, administration of tranexamic acid, or mechanical interventions such as uterine tamponade or uterine artery ligation [74,75].

## 3. Conclusions

Although pregnancy-related cardiac disease is best dealt with through a multidisciplinary team, emergency physicians must remain ready to independently manage unstable cases. A periodic review of potential illnesses and treatment strategies is essential for physician comfort during rare presentations of critically ill pregnant patients with cardiac emergencies.

## Figures and Tables

**Table 1:** Quick reference for cardiac emergencies in pregnancy.

Cardiac Condition	ED Presentation	ED Management
Gestational Hypertension, Pre-eclampsia, and Eclampsia	Hypertension, proteinuria, swelling, headache, visual disturbances, abdominal pain, seizures (in eclampsia)	Blood pressure control (IV labetalol or hydralazine preferred); magnesium for seizures.
Peripartum and Postpartum Cardiomyopathy	Shortness of breath, edema, fatigue, decreased exercise tolerance	Diuresis with furosemide; vasodilators such as hydralazine and nitrates. Avoid ACE inhibitors or ARBs. Ionotropes if needed.
Arrhythmias	Palpitations, dizziness, syncope	Arrhythmia-specific medications, cardiovert if unstable. Avoid amiodarone. Low molecular weight heparin preferred for anticoagulation; avoid warfarin in the first trimester.
Valvular Disease	Shortness of breath, fatigue, edema; can develop heart failure symptoms or arrhythmias	Management of concurrent heart failure or arrhythmias as above; anticoagulation with heparin if needed.
Aortopathies	Sudden severe chest and back pain, pulse deficits, may progress to tamponade	Blood pressure control (esmolol, labetalol), surgical consultation.
Congenital Heart Disease and Pulmonary Hypertension	Shortness of breath, fatigue, edema; heart failure symptoms	Oxygen, phenylephrine for hypotension, and cautious diuresis with furosemide. Avoid vasodilators. Inhaled nitric oxide and prostacyclins for pulmonary hypertension crises.
Coronary Artery Concerns	Chest pain, shortness of breath, diaphoresis, nausea	Aspirin, heparin, percutaenous coronary intervention.
Anticoagulation Issues and Bleeding	Swelling, pain, or redness in legs (for deep vein thrombosis). Chest pain, shortness of breath, and hemoptysis (for pulmonary embolism) Bleeding, bruising, or petechiae (for thrombocytopenia)	For deep vein thrombosis or pulmonary embolism, low-molecular-weight heparin is preferred over unfractionated heparin. Hemorrhage management requires a multidisciplinary approach, which may include transfusion, uterotonics (e.g., oxytocin), tranexamic acid, and mechanical interventions like uterine tamponade or artery ligation.

## Data Availability

Not applicable.

## Abbreviations

The following abbreviations are used in this manuscript:

ED	Emergency department.
PPCM	Peripartum/postpartum cardiomyopathy.
ECG	Electrocardiogram.
POCUS	Point-of-care ultrasound.
TTE	Transthoracic echocardiogram.
LVEF	Left ventricular ejection fraction.
LVEDD	Left ventricular end-diastolic dimension.
SVT	Supraventricular tachycardia.
VT	Ventricular tachycardia.
CHD	Congenital heart disease.
PH	Pulmonary hypertension.
ICU	Intensive care unit.
ACS	Acute coronary syndrome.
AMI	Acute myocardial infarction.
SCAD	Spontaneous coronary artery dissection.
PCI	Percutaenous coronary intervention.
STEMI	ST-segment elevation myocardial infarction.
DIC	Disseminated intravascular coagulation.
VTE	Venous thromboembolism.
DVT	Deep vein thrombosis.
PE	Pulmonary embolism.
HELLP	Hemolysis, elevated liver enzymes, low platelets.
